# A Systematic Review of Phenibut Withdrawals

**DOI:** 10.7759/cureus.68775

**Published:** 2024-09-06

**Authors:** Christopher Stewart, Hunter Simonsen, Savitha K Satyasi, Nauman Ashraf, Suporn Sukpraprut-Braaten

**Affiliations:** 1 Medicine, Kansas City University, Joplin, USA; 2 Psychiatry, Freeman Health System, Joplin, USA

**Keywords:** addiction, online supplement, phenibut, supplement abuse, withdrawal symptoms

## Abstract

Phenibut is an anxiolytic agent that was originally used as a treatment for anxiety, depression, post-traumatic stress disorder (PTSD), and insomnia. It is a gamma-aminobutyric acid (GABA) mimetic, which stimulates GABA receptors in the brain. This increases the inhibitory effects of GABA leading to a greater chance of a sedative response and risk for abuse. It is not currently registered in Western countries but is easily accessible online as a supplement. This is a systematic review of case reports of phenibut patients with withdrawal symptoms published in the PubMed database between January 2010 and October 2023. Following the inclusion criteria application, 15 articles were included. Descriptive statistics were used to analyze the results. The average age of patients with phenibut withdrawals was 31.8 years (SD=12.66 years), and 13 cases (87%) were males. The average dosage was 13.6 g/day (SD=8 g), ranging from 1.5 to 28.5 g/day. Nine cases (60%) presented at an emergency department, and three cases (17%) were presented at a clinic setting facility. The most common history of patients who took phenibut was alcohol or drug abuse (73%). A history of anxiety and depression (60%) was also seen in the majority of patient presentations. Phenibut is never prescribed in the United States, and there are no official guidelines for phenibut use. Educating all physicians about the potentially harmful supplements available to patients and their biological mechanisms is essential. This review highlights the importance of collecting a thorough patient history, including supplements, to help prevent phenibut misuse and subsequent withdrawals.

## Introduction and background

Overview of phenibut

Phenibut is an anxiolytic substance developed in Russia for the treatment of anxiety, depression, post-traumatic stress disorder (PTSD), and insomnia [1,2]. Initially designed as an anxiolytic agent for astronauts, it functions as a gamma-aminobutyric acid (GABA) mimetic, meaning it stimulates GABA receptors by increasing the influx of chloride ions through ligand-gated channels [2]. While phenibut targets both GABAB and GABAA receptors, it targets GABAB receptors to a much greater extent [2]. This causes a muscle-relaxing and a calming effect as GABA is the main inhibitory neurotransmitter of the CNS [2]. Although phenibut is not currently registered in Western countries, it is readily accessible online without a prescription as a supplement and is also marketed under various names including Anvifen, Fenibut, and Noofen on eBay and other online retailers [2-4]. Despite its efficacy in improving these conditions, users can quickly develop dependence [5]. 

Clinical challenges

Phenibut presents unique challenges in clinical settings. As a GABA agonist, symptoms of phenibut resemble those caused by substances such as benzodiazepines and barbiturates [6,7]. However, phenibut does not show up on any standard drug tests, and no commercial tests are available for its detection [8]. This complicates the diagnosis of phenibut abuse and dependence, particularly if patients do not disclose its use [8]. Since phenibut is often advertised as a supplement, patients may not consider its pharmaceutical properties and fail to mention it in their medical history.

Accessibility and risks

One challenge in addressing phenibut abuse is its marketing as a supplement [9]. Supplements are not approved by the Food and Drug Administration (FDA) and are legal to buy, sell, and use. Phenibut is more than a mere supplement as it has pharmacologic properties that necessitate regulation. If phenibut had FDA approval, it could be used in clinical settings for treatment. However, it does not have FDA approval due to addiction potential. Phenibut is a controlled substance in Australia and banned in the countries of Hungary, Italy, and Lithuania, further highlighting the danger of this substance [9]. Although there is limited research on phenibut’s potential for addiction, cases have been reported where patients develop a tolerance in as little as a week, indicating the potential danger and risks of taking this supplement [9]. While individuals may take this drug thinking they are using a supplement to help with their anxiety and depression, serious side effects may occur, especially when phenibut is combined with other substances, including coma and death [9,10]. Additional side effects that may occur when taking phenibut include relaxation, drowsiness, sedation, confusion, irritability, delirium, seizures, dilated pupils, high blood pressure, and increased heart rate [9]. 

Study aims

The primary aim of this study is to examine the reported cases of individuals presenting with phenibut dependence and to aid physicians in identifying patients to improve health outcomes. Additionally, this study seeks to educate physicians and raise awareness about substances marketed online as supplements that actually contain pharmacologic properties. Supplements contain vitamins, minerals, or other ingredients that an individual needs due to an insufficiency [11]. Phenibut falls outside of this scope. It is crucial for patients considering phenibut to understand the risks and potential for addiction, recognizing that they are not consuming a supplement. 

## Review

Methods

Search Strategy

This systematic review was conducted with case reports found in the PubMed database with the keyword “Phenibut.” A total of 25 cases published from 2010 to October 2023 were identified and obtained. All case reports were screened for patients using phenibut. The authors then extracted data on the reasons for taking phenibut, withdrawal symptoms, and treatments associated with phenibut abuse. *Inclusion Criteria*

**Figure 1:**
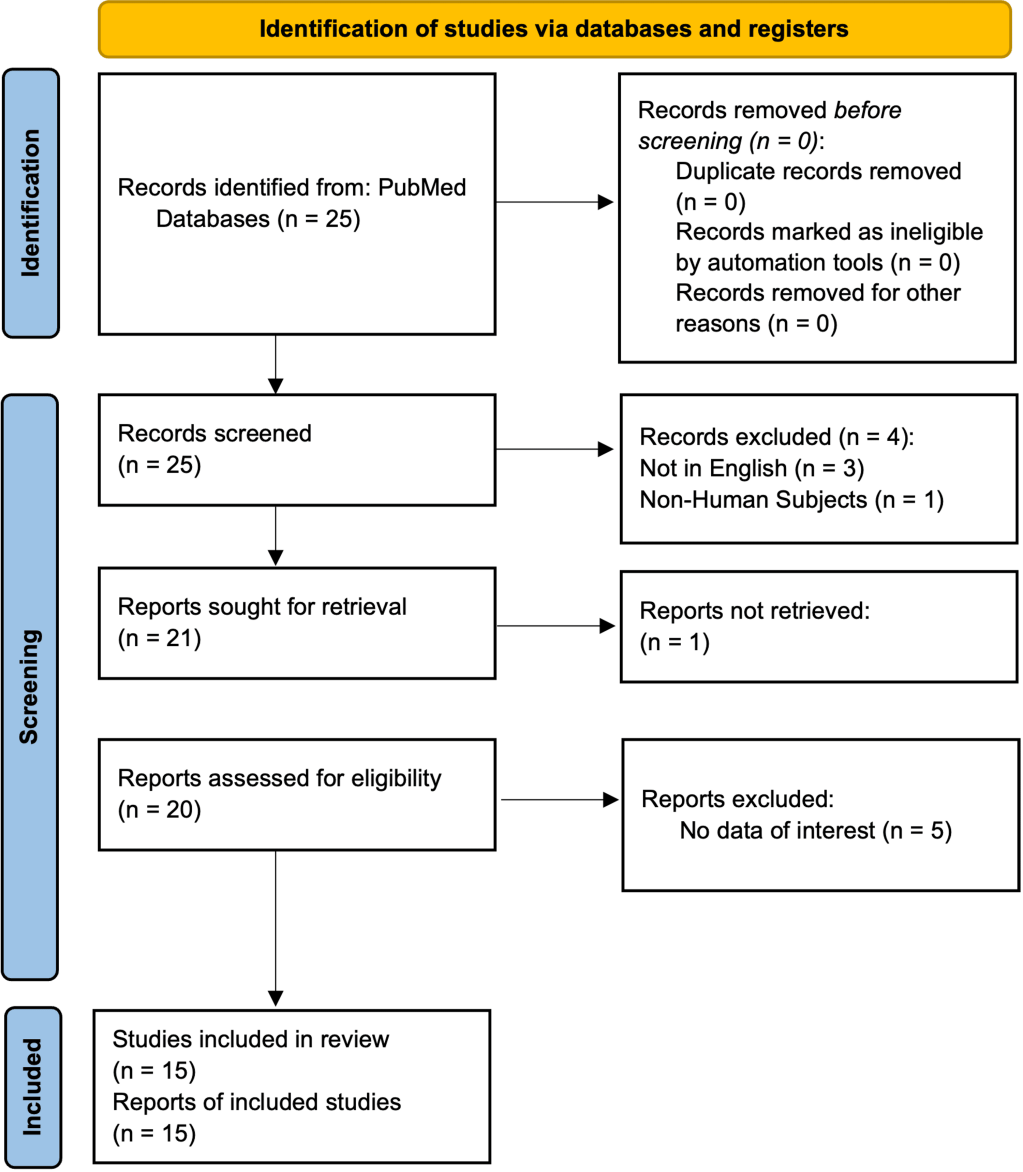
Preferred Reporting Items for Systematic Reviews and Meta-Analyses (PRISMA) Diagram

Inclusion criteria included patients presenting with withdrawal symptoms from phenibut ingestion. Various reports were excluded for the following reasons. One report was excluded because the subject was a canine, three were not in English, five were excluded because they focused on overdoses or irregular responses without detailing withdrawal symptoms, and one article could not be obtained. This selection process is illustrated in the Preferred Reporting Items for Systematic Reviews and Meta-Analyses (PRISMA) diagram shown in Figure 1.

Data Extraction

The population analyzed in this systematic review consisted of patients presenting with phenibut withdrawals. Data collected included the reasons for taking phenibut, the clinical settings in which they presented, the withdrawal symptoms they exhibited, and the methods used for detoxification. This data was compared to understand the motivations behind phenibut use, best practices for tapering patients off of phenibut, and common withdrawal symptoms. Understanding these aspects is particularly important because phenibut does not appear on standard drug screenings or blood tests. The difficulty in making a formal diagnosis highlights the need for increased awareness and consideration of phenibut abuse in differential diagnoses along with a thorough history taking.

Statistical Analysis

Statistical analyses implemented in this systematic review include descriptive statistics. Descriptive statistics was used due to the small sample size of cases that have been reported. Data are presented using tables and bar graphs, which are the most suitable formats for this type of data.

Patients were first grouped for comparative analysis based on the reasons for phenibut use. Two primary groups were identified: First, patients using phenibut to overcome substance misuse (alcohol, opioids, methamphetamines, other drugs), and second, patients using phenibut for psychological conditions (depression, anxiety, insomnia, etc.).

Next, patients were grouped according to the type of clinic in which they presented. This was done to understand the most common settings for phenibut presentation, which is relevant because phenibut misuse is difficult to diagnose using standard drug tests. Identifying common presentation settings helps target education efforts for physicians. The groups were emergency settings (emergency department) and outpatient clinics (family medicine, internal medicine, psychiatry).

Finally, patients were grouped based on the withdrawal symptoms they presented with. This helps physicians recognize phenibut withdrawal when other drug and blood screenings are normal. The withdrawal symptoms were categorized into self-harm, psychomotor agitation, and hallucinations. These categories were selected because they are common withdrawal symptoms and were the common symptoms seen in the reported cases. 

This systematic review aims to understand phenibut use by analyzing patient demographics, dosages, and durations, with a specific focus on withdrawal patterns. By examining reported cases of phenibut addiction, this review seeks to help physicians identify and manage phenibut use and raise awareness about the risks of substances like phenibut which are marketed as supplements but have pharmacologic properties.

Quality Assessment and Risk of Bias

The Joanna Briggs Institute for Case Reports (JBI) criteria were applied to case reports utilized in this review. This was essential to ensure that the case reports contained sufficient information to be accurate. The JBI criteria require that before using a case report the case report includes the patient’s demographic and characteristics, history, timeline, current clinical condition, and diagnostic tests and assessment, intervention/treatment is described, post-intervention clinical condition is described, adverse events/unanticipated events are identified and described, and the case report provides takeaway lessons. Potential bias in this review includes selection bias, in which the cases selected are not representative of all individuals using phenibut, and information bias, in which there are inaccuracies in the data reported. Information bias was prevented as best as possible by implementing the JBI criteria for case reports. Selection bias is possible due to the small sample size of case reports published on phenibut use.

Results

Of the 15 cases reported, 15 (100%) reported withdrawal symptoms and treatment. When looking at gender, 13 (87%) of the 15 cases were males and two (13%) were female. The average age of an individual with a phenibut addiction was 31.8 years (SD=12.66). Symptoms were categorized as either neurologic or physical. All 15 (100%) cases reported neurological symptoms and 11 (73%) reported both physical and neurological symptoms. The patients ingested an average of 13.6 g (SD=8 g) of phenibut each day for an average time period of 8.2 months. When looking at the location of the presentation, 9 (60%) of these cases were brought to the emergency department, whereas the remaining 6 (40%) were brought to psychiatric, general practice, or addiction medicine clinics. All individuals obtained phenibut either to help overcome other addictions (6, 40%), to help with anxiety (6, 40%), or to help with insomnia (3, 20%). When looking at tapering methods for withdrawal treatment, 9 (60%) were successfully treated with a Baclofen taper, with the majority of the remaining cases using benzodiazepine tapers (6, 40%). Table 1 shows the demographics, symptoms, and treatment for patients presenting with phenibut abuse. 

**Table 1:** Key Findings From Systematic Review y/o, years old.

Author (year)	Demographic	Reasons for taking	Presenting symptoms	Withdrawal symptoms	Dose (g/day)	Time on phenibut	Treatment
Samokhvalov (2013) [11]	35 y/o male	Stop alcohol, anxiety, and stress	Anger	Insomnia, restlessness	8	10 months	Baclofen taper
Hardman (2019) [12]	23 y/o male	Stop drugs	Hallucinations, psychomotor agitation	Hallucinations, tachycardia, myoclonus, hypertonic muscles	16	Not reported	Baclofen taper
Vandreese (2022) [13]	34 y/o male	Stop opioids	Agitation, sweating, hallucinations, confusion	Hallucinations, encephalopathy, twitching	28.5	36 months	Baclofen taper
Zheng (2019) [14]	23 y/o male	Stop alcohol	Suicidal ideation, seeking detoxification	Cold sweats, anxiety, insomnia, hallucinations	5	1 month	Benzodiazepine
Mash (2020) [15]	27 y/o male	Help relax	Hypertensive, found unconscious at home	Insomnia, restlessness, irritability, disorientation, heat flashes	15	Not reported	Benzodiazepine taper
Ahuja (2018) [16]	21 y/o male	Help with anxiety and school	Insomnia, hallucinations, anxiety	Hallucinations, anxiety	Not reported	Few months	Baclofen taper
Peterkin (2022) [17]	38 y/o female	Stop opioids	Jumped out of three-story window - trauma	Abdominal pain, headache, diaphoresis, lacrimation, restlessness, myalgia, anxiety, yawning, mydriasis, tachycardia	2	Not reported	Methadone and diazepam
Esposito (2021) [18]	50 y/o female	Help with anxiety and insomnia	Psychomotor agitation, insomnia	Abnormal motor behaviors, disorganized thinking, echolalia, visual hallucinations, insomnia	5	Several months	Baclofen taper
Joshi (2017) [19]	32 y/o male	Not reported	Suicide, insomnia, tachycardia	Tremors, hallucinations, insomnia	16	Not reported	Baclofen taper
Li (2017) [20]	24 y/o male	Not reported	Agitation, psychosis	Agitation, psychosis, respiratory failure	5	2 months	Not reported
McCabe (2019) [21]	27 y/o male	Help with anxiety	Self-harm	Agitation, tachycardia, hypertension, myoclonus, hallucinations	20	2 months	Baclofen and phenobarbital
Wainblat (2023) [22]	68 y/o male	Insomnia and anxiety	Seeking detoxification	Panic attack, insomnia, anxiety	7	Several months	Baclofen and phenobarbital
Brunner (2017) [23]	29 y/o male	Stop opioids. Not tested in drug test	Increased somnolence	Anxiety, tremors, craving, muscle aches	15	4 months	Phenobarbital
Coenen (2019) [1]	25 y/o male	Insomnia	Brought for detoxification	Changes in body temperature, depersonalization, hallucinations, insomnia, decreased appetite, sadness, irritability	34	24 months	Baclofen taper
Magsalin (2010) [5]	21 y/o male	Restless leg syndrome	Anxiety	Anxiety, tremors, fatigue, psychomotor agitation, irritability, tachycardia, insomnia, decreased appetite	1	2 weeks	Phenibut taper

The common presenting symptoms were isolated from the data. Self-harm (4, 27%) and psychomotor agitation (4, 27%) were listed as the most common withdrawal symptoms, followed by hallucinations (3, 20%), seeking detoxification (3, 20%), and insomnia (3, 20%). This can be seen in Table 2.

**Table 2:** Most Common Presenting Symptoms in Phenibut Patients

Presenting symptom	Number (%)	Average dose of phenibut in grams (standard deviation)
Psychomotor agitation	4 (27%)	13.6 (11.2)
Self-harm	4 (27%)	10.8 (8.6)
Hallucinations	3 (20%)	22.3 (8.8)
Seeking detoxification	3 (20%)	15.3 (16.2)
Insomnia	3 (20%)	10.5 (7.8)
Tachycardia	2 (13%)	15.5 (0.7)
Anxiety	2 (13%)	Not reported

The most common withdrawal symptoms can be found in Table 3, and were insomnia (8, 53%), hallucinations (8, 53%), and muscle abnormalities (8, 53%). Note that the percentages add up to more than 100% as many patients reported multiple symptoms. 

**Table 3:** Most Common Withdrawal Symptoms in Phenibut Patients

Withdrawal symptom	Number (%)	Average dose of phenibut in grams (standard deviation)
Insomnia	8 (53%)	11.4 (10.5)
Hallucinations	8 (53%)	17.8 (11.0)
Muscle abnormalities	8 (53%)	12.9 (9.6)
Anxiety	6 (40%)	6 (5.5)
Restlessness	3 (20%)	8.3 (6.5)
Tachycardia	3 (20%)	12.7 (9.5)
Irritability	3 (20%)	16.7 (16.6)
Disoriented	2 (13%)	10 (7.1)
Agitation	2 (13%)	12.5 (10.6)

**Figure 2:**
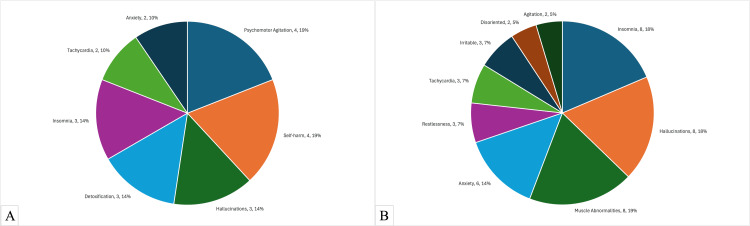
Comparison of Withdrawal and Presenting Symptoms A. Withdrawal Symptoms. B. Presenting Symptoms.

Figure 2 shows a side-by-side comparison of withdrawal and presenting symptoms. Note that the percentages shown in Figure 2 are different from the percentages in Tables 2, 3 because of Tables 2, 3 factoring in several patients presenting with multiple symptoms. Figure 2 looks at the total number of symptoms reported and calculates the percentage based on this number.

**Figure 3:**
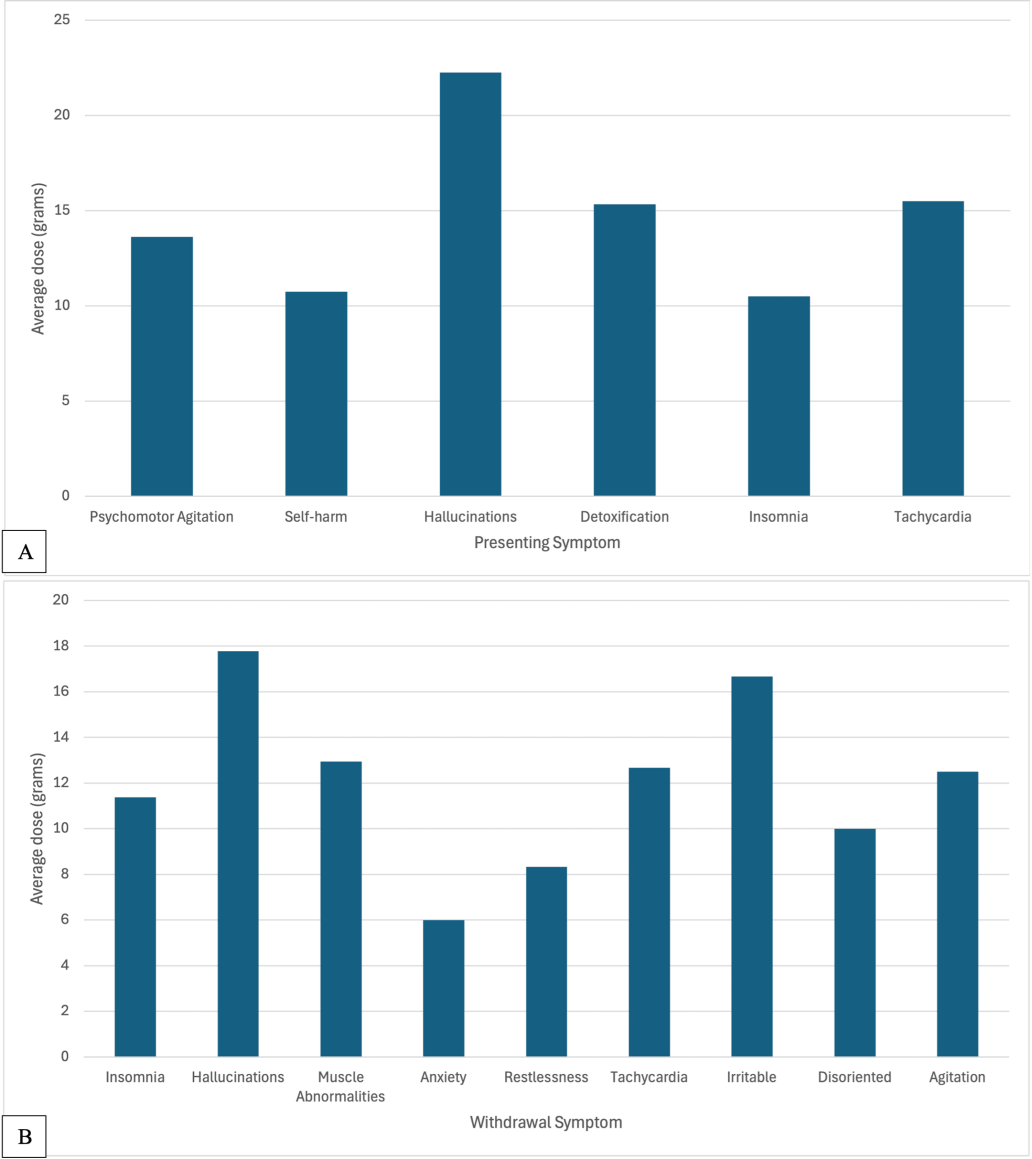
Comparison of Symptoms by Dose A. Presenting Symptoms by Dose. B. Withdrawal Symptoms by Dose.

Next, an analysis was done on the dose of phenibut taken with each reported symptom. Figure 3 compares the dosages for presenting and withdrawal symptoms. 

Finally, of the cases reported, 11 (73%) patients were taking phenibut to help overcome alcohol or drug misuse while 9 (60%) were taking phenibut as an anxiolytic and antidepressant. These add to over 100% because some patients listed multiple reasons for taking phenibut. 

Discussion

Introduction and Relevance

Phenibut is a legally available substance with pharmacologic properties similar to prescription medications (GABA agonist). It is inexpensive and available online [10,24,25]. It is important that awareness of this substance is increased, methods for withdrawal are evaluated, and the mechanism of phenibut is understood to increase the quality of care received by addicted patients or patients seeking medical care. As mentioned, phenibut does not appear on standard drug tests and there is no commercial test available, posing a diagnostic challenge for physicians. Proper history taking is essential, and physicians should specifically inquire about supplement use, as patients may not consider phenibut alongside prescription or OTC drugs. Physicians should keep phenibut on their differential when encountering patients with alcohol-like withdrawal symptoms and negative test results.

Physician Education and Raising Awareness

Educating physicians about supplements that carry a risk for harm, including their biological mechanisms, is crucial to determining methods of prevention and treatment. Phenibut should be considered in differential diagnoses for patients presenting with withdrawal symptoms like self-harm, psychomotor agitation, and hallucinations, even when standard tests are negative. Physicians are also key to raising awareness among patients and the community of what manufacturers often advertise as supplements but contain pharmacologic properties. Increased awareness of online supplements is vital, as these products have significant pharmacologic properties and addiction potential. Besides phenibut, other concerning supplements include kratom (stimulant) and tianeptine (NMDA agonist). Patients may quickly develop dependencies on these substances.

Tapering Strategies

Common strategies for tapering phenibut addiction include using baclofen and benzodiazepines. Baclofen, a GABA agonist and muscle relaxant, is effective in treating alcohol use disorder [26,27]. It is used for tapering phenibut due to its ability to activate the same GABA receptors, eliciting similar responses. Gradual dose reduction helps mitigate withdrawal symptoms. While used less often in reported cases, benzodiazepines, methadone, diazepam, and phenobarbital were also successful in mitigating withdrawal symptoms. 

Mechanism of Action

Phenibut functions as a GABA agonist due to its structural similarity to GABA, featuring an additional phenyl ring and carboxyl group [2]. This structural modification enhances its lipophilicity, enabling it to cross the blood-brain barrier and exert potent effects [4]. Activation of GABA receptors increases the effects of the inhibitory neurotransmitter GABA, resulting in calming and anxiolytic effects [4].

Demographic Trends

Based on the results previously discussed, phenibut abuse shows to be more prevalent in younger males. This finding helps us identify the population that is most likely to be affected by this drug. Physicians should be aware of phenibut use as a possible diagnosis, especially when presented with a male patient between the ages of 20 and 35 years with withdrawal-like symptoms and negative drug screening. From cases reported, women may not be at as high of a risk as males as they accounted for a much smaller portion of the patient population reviewed.

Higher doses of phenibut were also seen in patients with addictive behavior and more associated symptoms. On average, patients consumed significantly more than recommended, which gives another factor to be aware of when taking a medical history. Because phenibut is a supplement and not a drug, there is no FDA-approved dosage of phenibut; however, guidelines generally recommend 500-1500 mg per day, which is significantly lower than the consumption of the patients who were reviewed [28,29]. This underscores the importance of thorough history-taking, as patients may be unaware of their addiction. Physicians should inquire about all supplements to accurately assess intake as well as the amount of each supplement. 

Limitations and Future Directions

This study's limitations include the small number of cases available, along with the limited age range and the gender of reporting patients. Another limitation of this study is that the study is based on cases that have been reported. The doses reported are based on the dosages reported by the patient. Further, we do not know exactly where the patients obtained their phenibut. Quality factors such as the purity of phenibut are not known and could contribute. These are all factors that we are unable to account for. This study focused on the limited data that are available to increase awareness and health outcomes. Increasing the reporting of phenibut cases will enhance the available data for review. As more information becomes available on phenibut addiction and withdrawal, our understanding of this drug will improve.

## Conclusions

Phenibut is an easily accessible and legal substance with prescription-grade pharmacologic properties (GABA agonist) that does not show up on drug tests. Awareness of the drug adds differential diagnosis to physicians who see individuals with alcohol-like withdrawals, but all tests are negative. It is important to educate all physicians about potentially harmful supplements that are available to patients and their biological mechanisms. This review highlights the importance of collecting a thorough patient history, including supplements, to help prevent phenibut misuse and subsequent withdrawals. This review further educates physicians on how phenibut withdrawals can be treated, and to be wary of the facade many online retailers market as supplements.
